# The Pharmaceutical Industry’s Future: How Artificial Intelligence is Transforming Medicine

**DOI:** 10.34172/apb.025.45904

**Published:** 2025-09-10

**Authors:** Barnika Debnath, Samson K Wilson, Subhrajyoty Basu, Sai Yasasvi Kompella, Rakesh Singha, Santosh Kumar Sahoo, Nitin Yadav, Partha Roy, Amit Kundu

**Affiliations:** ^1^Department of Pharmaceutical Analysis, School of Pharmacy, GITAM(Deemed to be University, Visakhapatnam, India; ^2^Department of Pharmacology, School of Pharmacy, GITAM(Deemed to be University, Visakhapatnam, India; ^3^Department of Pharmaceutical Chemistry, School of Pharmacy, GITAM(Deemed to be University, Visakhapatnam, India

**Keywords:** Artificial intelligence, Machine learning, Pharma sector, Nanobots, Drug repurposing, Deep learning

## Abstract

In the pharmaceutical industry, artificial intelligence (AI) is revolutionizing individualized therapy, research, and drug development. AI includes machine learning (ML) and deep learning (DL), that are used to read enormous amounts of data, spot mysterious patterns, and find possible medication candidates more quickly. AI is also improving clinical trials through better patient recruitment, real-time data monitoring, and trial outcome prediction. It also customizes care according on a person’s genetic composition, lifestyle, and environmental factors is also supporting personalized medicine, a novel approach to healthcare. In, pharmaceutical industries it is used to simplify medicine production procedures, enhancing quality control, and streamlining supply chain management that saves the valuable time as well as billions of dollars. This comprehensive review discusses the different impacts of AI-enabled technologies on each stage of the pharmaceutical life cycle. It demonstrates that ML, data analytics and predictive modelling can accelerate drug discovery, improve manufacturing processes, streamline quality processes, enhance formulation approaches, and transform post-marketing surveillance, drug repurposing, precision medicine, and nanobots.

## Introduction

 One of the world’s most research-intensive industries, the pharmaceutical sector consistently produces cutting-edge medications that enhance and save lives.^1^ Traditional drug discovery is time intensive and resource demanding, often relying on interactive screening. The advent of new technologies such as machine learning (ML) is reshaping the conventional processes of drug discovery, development, and even the entire lifecycle management in the pharmaceutical industry.^2^

 Artificial intelligence (AI) combines various smart behaviours and processes. These are created by computer models, algorithms, or a set of rules. They allow machines to mimic human thinking skills such as learning and problem-solving.^3^ In general we can conclude the process of mimicking systems that behave like humans is called AI. ML is a part of AI. It works by using data-trained algorithms. Deep learning (DL) is a subset of ML that somewhat based on how the human brain is structured.^4^ AI, ML and DL are increasingly pivotal in pharmaceutical data analytics and automation.^1^ This review discusses about the impact of AI around drug discovery, development, supply chain management, quality control and quality assurance pharmacovigilance, nanobots, personalised medicine.^3^

## Machine learning

**Figure 1:**
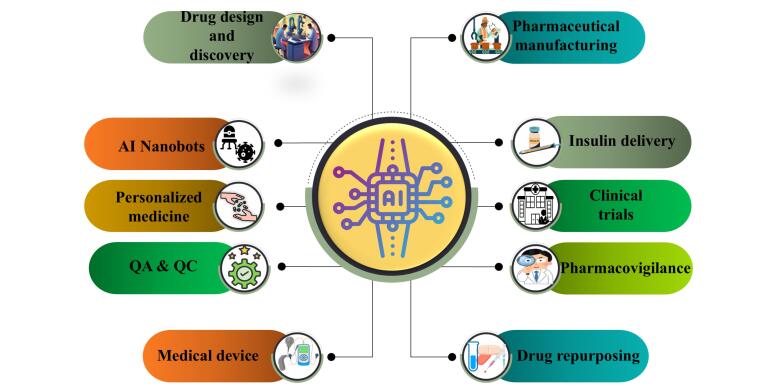
Classification of AI

 A branch of AI and computer science known as ML focuses on algorithms and data to simulate human learning and slowly enhance the accuracy of AI, as shown in Figure 1. Significant opportunities arise from integrating proteomic and genomic analytics with AI algorithms for drug development.^5^

 ML enhances drug discovery by aiding in repurposing, drug-protein interaction prediction, efficacy assessment, and toxicity prediction. It streamlines R&D, reduces costs, increases lead optimization, and lessens animal testing, while improving patient compliance and treatment efficiency using real-world data.^6^

 Types of ML includes (as shown in Figure 1):

###  Supervised learning

 Supervised learning uses labelled data to train algorithms for prediction and classification tasks. It identifies relationships between input variables and a target output, enabling models to make accurate, task-specific decisions based on verified data and explicit guidance for determining outcomes.^5^

 In pharmaceutical industry supervised learning is used to predict drug efficacy and key pharmacokinetic properties, including absorption, distribution, metabolism, and excretion (ADME). These models are typically trained on large datasets containing thousands of drug compounds, utilizing molecular descriptors like logP and molecular weight as input features. They mainly classify a drug’s metabolic stability by understanding its accuracy rates frequently surpassing 80%. This predictive strength is especially valuable in the early stages of drug development, where it helps streamline the process by identifying high-potential candidates, and reduce the costs.^7^

 Supervised learning comprises of some significant techniques such as support vector machines (SVMs), k-nearest neighbours (kNNs), Naïve Bayes, Decision Trees, AdaBoost, Random Forests, which is as shown in Table 1, Figure 1.

**Table 1:** Explanation of different techniques of supervised learning^6^

**Supervised learning techniques**	**Description**	**Application**	**Reference**
SVMs	SVMs have demonstrated strong performance in virtual screening by effectively identifying structurally diverse compounds with similar biological activity.	Novel chemical series that are active against GPCR s have been successfully predicted by SVMs utilizing customized training and validation datasets. They have also been used recently in precision psychiatry to diagnose mental illnesses like Alzheimer's, schizophrenia, and depression.	^8,9^
kNNs	It is commonly applied in disease prediction. kNNs’ algorithms, have drawn increased attention in bioinformatic tasks, notably vaccination target prediction.	The kNNs algorithm is used to identify significant patterns related to prescribing activities such as class of drugs, doses that are prescribed, and treatment procedure. Utilizing ML algorithms such as KNN, in healthcare systems it helps in improving decision-making and improve efficiencies.	^10,11^
Naïve Bayes	The Naive Bayes classifier assigns the class with the highest likelihood as the most likely outcome by using Bayes' theorem to predict the probability of input data belonging to a class based on independent attributes.	Naïve bayes is used as a prediction model for myelotoxicity by drug induction. In one of the study is shows overall prediction accuracy is 94% for the training set and for the external test set.	^12,13^
Decision trees	Decision trees are effective visual tools for risk analysis and decision-making, easily understood without complex math knowledge.	Decision trees are used in pharmaceutical QSAR predictions, handling diverse datasets and providing clear, interpretable outputs for complex, nonlinear relationships.	^14,15^
AdaBoost	AdaBoost enhances classification by combining weak learners into a strong model. Its improved version treats drug-protein interaction prediction as a classification task, achieving higher accuracy in matrix completion on public datasets.	Drug-protein prediction is typically approached as a matrix completion or scoring task. We enhanced the AdaBoost algorithm to reframe this problem as a classification task, improving prediction performance.	^16,17^
Random forests	Random Forests facilitate feature selection, classification, and regression in drug discovery. They improve ligand-protein affinity prediction, handle incomplete data, and need little tuning. RFs also enhance genomic analysis and drug response prediction, boosting virtual screening accuracy.	Random Forests have shown superior prediction, effectively classifying monoclonal antibodies with over 70% subcutaneous bioavailability. In oral bioavailability studies, a simplified RF model outperformed other ML methods by accurately distinguishing compounds with high or low absorption.	^5,18^

SVM, support vector machine; kNN, k-nearest neighbour; RF, Random forest; ML, machine learning; GPCR, G-Protein coupled receptors; QSAR, Quality Structure activity relationships.

 Unsupervised learning comprises of some significant techniques such as clustering algorithms, dimensionality reduction, anomaly detection, association rule mining, topic modelling as shown in Table 2.

**Table 2:** The description and application of different techniques under unsupervised learning

**Unsupervised learning techniques**	**Description **	**Application**	**Reference**
Clustering algorithms	Clustering methods group data points based on inherent similarities, revealing natural structures in complex datasets. They are unsupervised in nature and help uncover hidden patterns without prior labelling.	Used for analysing chemical structures, gene expression profiles, and patient datasets to enable target identification and disease classification.	^6,16^
Dimensionality reduction	These techniques reduce the number of variables in high-dimensional datasets while preserving the essential information, enhancing visualization and interpretation.	Applied in analysing imaging data, drug activity datasets, and gene expression profiles to extract critical features and enable informed decisions.	^19,20^
Anomaly detection	Anomaly detection identifies unusual data points that significantly deviate from expected behaviour. This helps highlight safety issues or data quality concerns.	Used in detecting adverse drug reactions, data entry errors, or unusual trends in clinical data for pharmacovigilance.	^21,22^
Association Rule Mining	A method to find significant relationships or co-occurrences between variables in large datasets. It helps generate interpretable rules from raw data.	Supports identification of drug-drug interactions, co-prescription patterns, and analysis of adverse event reports.	^23,6^
Topic Modelling	Topic modelling techniques extract hidden thematic structures from large volumes of unstructured text using probabilistic models like LDA.	Used to mine scientific literature, clinical reports, and social media to uncover research trends, patient opinions, or emerging fields.	^24,25^

###  Deep learning

 DL, a subset of ML, uses multiple hierarchical layers to process data, where each layer builds on simpler abstractions from the previous one. These layers apply linear and nonlinear transformations to extract features at various levels. Models like long short-term memory (LSTM), generative adversarial networks (GANs), and deep convolutional neural networks (CNNs) enable understanding of complex patterns directly from raw data, allowing high-level abstraction and powerful data representation, as shown in Figure 1.^26^

####  Generative adversarial networks 

 In the pharmaceutical product development industry, generative adversarial networks are often used to create novel chemical structures and thereafter improve their properties. The GANs architecture consists of a discriminative network that assesses the quality of the new molecular entities and a generative network that synthesizes these, which makes possible the development of structural diversity and functionally optimized drug candidates, as shown in Figure 1.^27^

####  Long short-term memory 

 LSTMs are a subtype of recurrent neural networks characterized by their excellent ability to recognize and forecast temporal correlations. They have found application in pharmacokinetics and pharmacodynamics, assisting in predicting drug concentration-time curves and evaluating the efficacy of drugs as shown in Figure 1.^28^

####  Convolutional neural networks 

 Inspired by the anatomical organisation of the visual cortex in animals, this advanced class of DL architectures was created especially for the processing of data with a grid-like arrangement, like visual representations. Convolutional, pooling, and fully connected layers are the three basic layers (or core components) that are typically used to form CNNs, which are essentially mathematical constructs. While the final layer, the fully connected layer, is in charge of converting the extracted features into the final output, like classification results, the first two layers, the convolutional and pooling layers, are mostly involved in the feature extraction process as shown in Figure 1.^29^

####  Recurrent neural networks (RNNs)

 These are DL models ideal for sequential data, retaining past input context. They are used in time series forecasting, speech recognition, and NLP, and in bioinformatics for analysing protein, RNA, and DNA sequences, aiding in gene prediction and protein structure analysis as shown in Figure 1.^30^

####  Autoencoder 

 Is an auto-associative neural network that mimics its input, having an output which is identical to the input. The AE network employs a set of recognition weights to transform an input vector into a code vector.^17^ From the code vector, a rough reconstruction of the original input is then reconstructed by a second set of generating weights. This basic AE serves as the foundation for training deep networks, allowing independent training of each layer of the deep network while leveraging autoencoding principles as shown in Figure 1.^31^

####  Artificial neural networks (ANNs)

 It was designed based on how the human brain operates. Such networks consist of processing units linked together in an artificial neurone, referred to at times as nodes or perceptron’s, which receive inputs and generate outputs accordingly.^18^ This process involves weighting and aggregating the inputs through an activation function, followed by computing the outputs using a predefined transfer function. By passing information through multiple neurons, ANNs enable the transformation of inputs into the final output as shown in Figure 1.^32^

## Applications of AI in pharmaceutical sector

**Figure 2:**
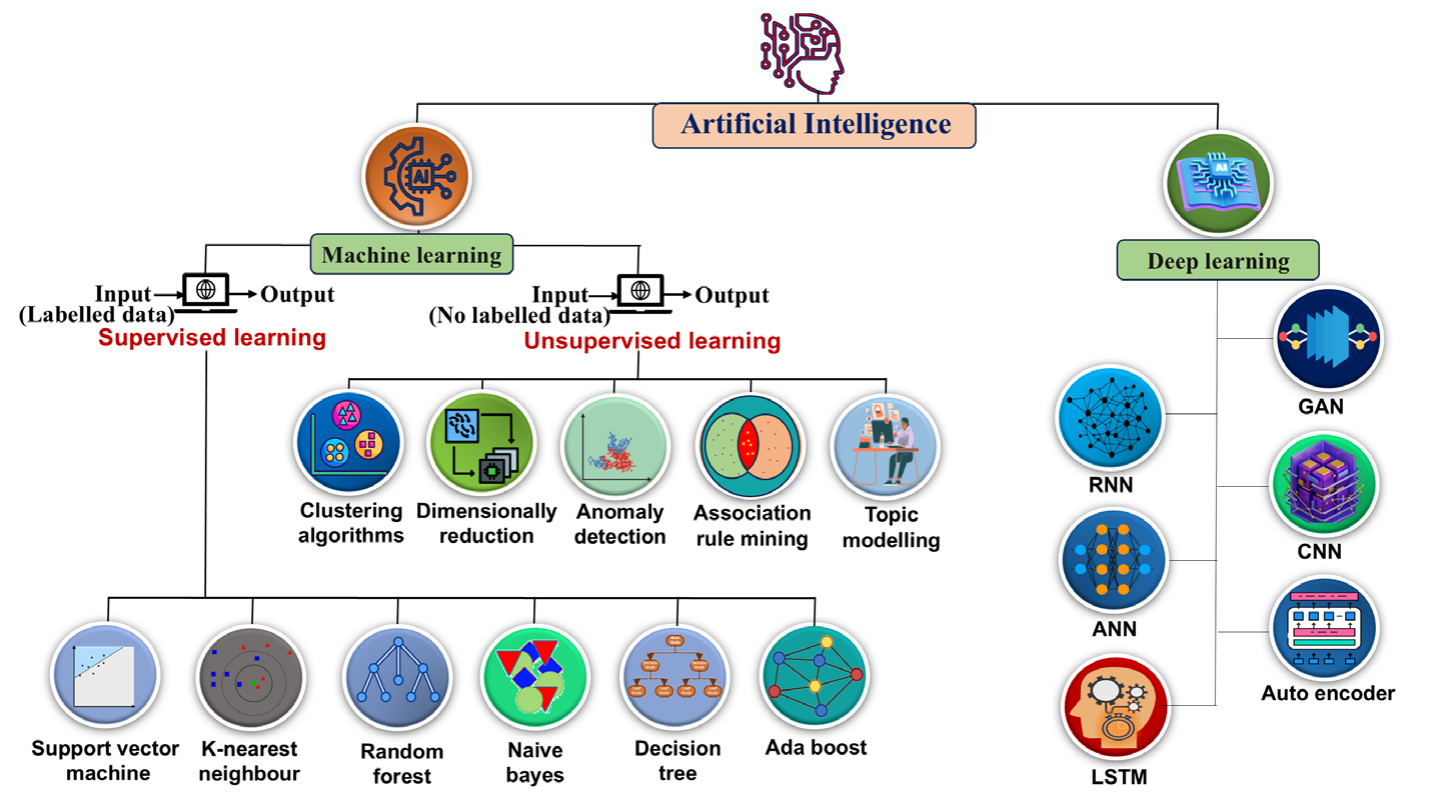
Application of AI in different pharmaceutical domain

 The application of AI in the pharmaceutical and healthcare sectors encompasses a wide range of areas, which includes: drug discovery and development, pharmaceutical manufacturing, insulin development, nanobots, personalised medicine, clinical application, quality control and quality assurance, pharmacovigilance, gene biomarker, drug repurposing as shown in Figure 2.^33^

###  AI in drug design and discovery

 By dramatically speeding up the process of finding and developing novel therapies, from fundamental research to the right candidate, AI is transforming the drug discovery industry. Conventional drug discovery can be time consuming and resources demanding relying on iterative screening.^34^ Algorithms based on AI can, however, analyse such huge datasets as proteomic, clinical, and genomic data rapidly to predict the efficacy of drug candidates, as well as to find potential targets for the drugs, as shown in Figure 2.

 The first step in the drug development process is the identification of new molecular targets. AI has demonstrated good potential in the very first stage of the drug discovery process that is the de novo drug design. The next step in the process is determining the kinetic parameters of binding to a specified target, its affinity, and other relevant characteristics. ^35^

####  Target identification

**Figure 3:**
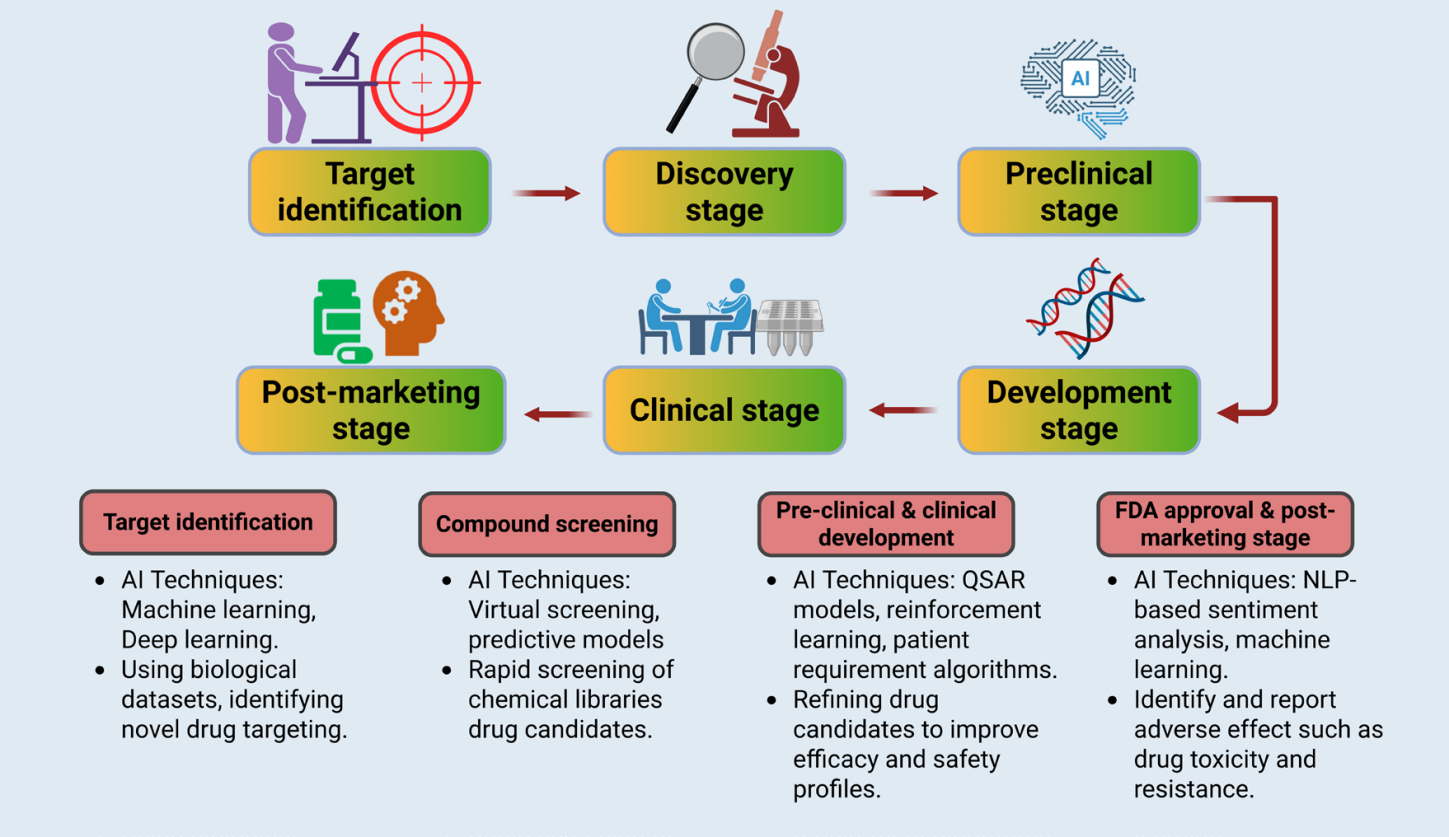
AI in drug discovery

 Target identification in drug development involves finding proteins or molecules whose activity changes disease states, as shown in Figure 3. ML models use genomic, proteomic, and interaction data to identify likely targets. DL platforms like DeepChem and AlphaFold2, supported by databases such as PubChem and ChEMBL, enhance this process, as mentioned in Table 3. Graph-based methods and GNNs uncover the causal links between gene and disease. A decision tree meta-classifier trained on network data—including transcription, metabolism, and localization—has been used to predict druggable genes, aiding precision in early-stage drug discovery.^34,36^

**Table 3:** Some examples of existing database used in the drug discovery and development^37^

**Database**	**Description**
PubChem	A free public dataset consisting of large information regarding chemical and bioactivity
ChemIDplus	An open access resource containing standardized chemical structure and nomenclature flies.
DrugBank	A large collection of drug’s pharmacokinetics, pharmacodynamics, mechanism of action of the drug, uses, side effects.
SIDER	It consists of marketed medicine and its adverse drug reaction.
Kyoto Encyclopedia of genes and genomes (KEGG)	A database consists of manually illustrated about the encapsulate molecular interactions and reactions for drug development
BindingDB	It consists of information about the binding affinity between drug and the target.
Manually Annotated Targets and Drugs Online Resource (MATADOR)	The dataset consists of medical indications, adverse effects, drug metabolization, pathways, and gene ontology
Therapeutic target database (TTD)	A database consists of therapeutic target between protein and nucleic acid.
Human microRNA disease database (HMDD)	A database that curated experiment-supported evidence for human microRNA (miRNA) and disease associations
GDSC	A dataset consists of drug responses and genomic biomarker

####  Screening of the compound with the help of AI

 AI can forecast the interaction between medications and proteins, approximate the bioactivities of compounds, and assist with virtual screening and optimisation. Predictive models that can identify compounds that are most likely to bind to a target protein is one way AI performs virtual screening. The models are trained on different types of data, including molecular descriptors, structural information, and known protein-ligand complexes, as per Figure 3.^34,36^

####  Pre-clinical and clinical development 

 Predicting potential drug responses is a vital component of the drug design process. Similarity-based or feature-based ML methods can be employed to forecast the effect of drug on individual cells and to assess the efficacy of drug-target interactions based on binding affinity or free energy of binding, as explained in Figure 3. Similarity methods operate on the premise that drugs with similar characteristics target similar proteins, while feature-based methods identify specific features of drugs and targets, which are then input into a classifier as a drug-target feature vector. DL approaches, such as DeepConv-DTI and DeepAffinity, exemplify these methods, as they learn embeddings for drugs and targets using convolutional and attention mechanisms.^34^

####  AI in FDA approval and post-marketing surveillance

 Natural language processing (NLP) can be applied to analyze scientific literature to identify and report adverse effects, such as drug toxicity or resistance, and generate automated assessments for regulatory approvals (like FDA) or patent submissions. Additionally, NLP-based sentiment analysis techniques can assist in recommending drugs, as shown in Figure 3. ML systems can also predict potential product sales, enabling pharmaceutical companies to better allocate and optimize their business resources. ^34^

####  Case Study

 With an overview of the AI software in drug discovery, let’s now consider real-world applications. The following case studies illustrate the significant impact these technologies have had in the field.

 The case study “BenevolentAI – Using AI to Disrupt the Traditional Drug Discovery Process” showcases how UK-based BenevolentAI uses its AI platform, Bioscience Machine Brain (BMB), to revolutionize drug discovery. By cutting early-phase and preclinical testing from 3–6 years to 1–2 years and reducing costs by 60%, BAI streamlines development and boosts efficiency. The platform rapidly identified promising compounds for ALS, now in clinical trials. The study explores BAI’s potential to disrupt the pharmaceutical industry amid growing AI competition. As health-tech startups and pharma giants embrace AI, significant progress in treating severe diseases is becoming increasingly achievable.

###  AI in clinical trials

 Patient recruitment and retention remain critical challenges in the clinical trial process, contributing to significant delays and increased costs. Recruitment alone can consume up to 30% of clinical trial timelines and costs, often leading to extended delays and financial losses. AI has emerged as a transformative tool to address these issues, streamlining patient identification and engagement through advanced data analytics. AI algorithms can analyse vast repositories of electronic health records, genomic data, and clinical histories to identify potential participants who meet the eligibility criteria, as shown in Figure 2.^38^

 Clinical trials for Alzheimer’s disease encounter several difficulties, such as unequal participant distribution and high screen failure rates. These issues can be effectively resolved by artificial in AI, which is explained by expanding volume and complexity of biological data.^39^

 Without the need for explicit programming, ML algorithms can improve predictive accuracy by revealing hidden patterns in data. In one study, 321 ADNI participants with baseline AT(N) biomarkers were analysed using a DL model to differentiate between rapid and slow disease progression. This was accomplished by grouping patients according to the pattern of their disease progression over time using an unsupervised time-series technique called dynamic time warping (DTW) in conjunction with Ward’s linkage clustering.^39^

 AI is being used by Alto Neuroscience, a 2019 startup, to create brain biomarkers for focused mental health therapies. ALTO-100, ALTO-202, and ALTO-300 are among the several medications for MDD and PTSD that are undergoing clinical trials. Patients with AI-identified biomarkers had greater response rates (61% vs. 33%), according to positive Phase IIa results for ALTO-100. Through partnerships and funding rounds, Alto has raised more than $100 million to further its AI-driven drug development.^40^

###  Al in pharmacovigilance

 AI systems integrate diverse data sources—like EHRs, social media, and patient forums—to identify adverse drug reactions (ADRs). This comprehensive approach captures patient experiences often missed by traditional methods, enhancing drug safety monitoring and post-market surveillance.^41^ There will be unstructured data like social media comments or patient discussions in forums. AI employs NLP to extract this data to analyse such unstructured data. It identifies different patterns and signals related to ADRs usually overlooked by traditional methods.^42^ Pattern recognition uses ML algorithms designed to recognize patterns in large datasets. Real-time monitoring with the help of AI leads to the immediate detection of ADRs as they occur, and facilitates quick responses to potential safety issues. ADR detection is more effective with AI as it relies mainly on the quality and quantity of the data it processes, as shown in Figure 2.^43^

 Pharmacovigilance is being revolutionized by a number of AI-powered tools that improve adverse event detection and drug safety monitoring for example AstraZeneca’s which uses AI-based system to detect adverse events and support regulatory compliance, but it requires skilled personnel and may miss rare events.119 IBM Watson for Drug Safety uses ML and NLP to analyze a variety of structured and unstructured data, improving decision-making. Adverse Health Analytics used SignalMine to monitor adverse event and risk assessment with increased efficiency. Oracle’s Argus Safety uses AI to automate signal detection and adverse event reporting.^44^

###  AI in pharmaceutical manufacturing

 Advancements in pharmaceutical manufacturing are increasingly driven by intelligent systems designed to replicate human expertise in response to rising process complexity and demands for efficiency and quality. Many of the pharmaceutical processes we use today are capable of being automated using more advanced methodologies such as computational fluid dynamics (CFD), which employs Reynolds-Averaged Navier-Stokes solvers to simulate agitation and stress in process equipment including stirred tanks. The flow-related challenges, such as turbulence, can also be addressed with techniques such as large eddy simulations and direct numerical simulations. In solid dosage forms, such as tablets, AIcan help developers optimize formulations by evaluating critical parameters during formulation development.^6^

 AI can also help predict and evaluate the physicochemical stability of oral formulations by reviewing vast data sets of drug properties, formulation variables, and environmental factors. For parenteral, transdermal, and mucosal drug delivery systems, AI can help optimize development workflows by predicting the behaviour of formulations and automation can enhance TNM parameters, including pH, solubility and stability, as well as viscosity.^45^

 AI can also help track constituents or particulates in formulations and provide recommendations on inspection instruments and timelines in order to deliver realistic quality assessments. When it comes to biological products, AI can help design proteins, peptides and nucleic acid therapeutics with improved properties, leveraging databases on protein structures and functions, to design models for optimizing therapeutic safety, efficacy, and immunogenicity. These models aid in maximizing immunogenicity, safety, and effectiveness of treatments, as shown in Figure 2.^45^

###  Use of AI in medical device

 AI used in medical devices with sophisticated data analysis and automation capabilities is revolutionizing healthcare. It makes diagnosis and treatment quicker and more precise, particularly for complicated neurological disorders like stroke, Alzheimer’s, and epilepsy.^46^ AI systems can effectively process the massive amounts of data generated by traditional tools like MRI and EEG to identify abnormalities in the nervous system. DL algorithms enhance medical imaging analysis and image recognition, including the detection of liver disease via ultrasound and radiology. By recording real-time cellular signals, AI also aids in vitro diagnostics, improving treatment results and diagnostic accuracy, as per Figure 2.^46^

 The operation of smart wearables, which are being utilized more in the fields of healthcare, sports, rehabilitation, entertainment, and smart home monitoring, depends heavily on AI and ML. Because of its accessibility and ease of use, the wrist is the preferred location for these devices. Smart wristbands are used in healthcare to track cardiovascular activity, diabetes, and heart failure. In one study it shows using sensor data analysed by ML, created a wrist-worn fall detection device for the elderly that achieved 91% accuracy in order to identify atrial fibrillation.^47^

**Figure 4:**
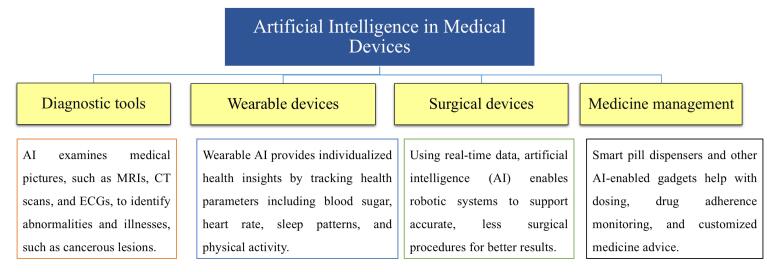
AI in medical device

 AI used in diverse domain such as diagnostic tools, wearable device, surgical devices, medical management, as shown in Figure 4.

###  AI in nanobots

 Nanobots robots that are just a few nanometres in width—provide fantastic promise in drug delivery because nanotechnology enables drugs to be delivered to specific sites, thereby decreasing side effects and enhancing efficacy^48^ (as per Figure 2). Nanomedicine uses nanorobots (0.5–3 μm) made of carbon materials to navigate capillaries, detect tissue damage, and deliver targeted therapies. These robots can be tracked by doctors to ensure they reach specific sites, such as tumours, for treatment.^49^

 Creating nanorobots requires advances in molecular manufacturing, including programmable diamond like structures for strength. While proteins excel at self-assembly, diamond-based nanomachines promise superior performance. Within 10–20 years, these tools may become standard in medicine, offering physicians powerful means to fight disease, aging, and poor health.^49^

 The electrical characteristics of muscle, fat, and skin tissue will all be reviewed to be able to maximize biosensors operation with in-body antennas without sacrificing data transmission. Nano-biosensors that could be used with a nanorobot may rely on cutting-edge materials such as functionalized carbon nanotubes (CNTs), where the protective layer will help provide longevity for the entire device. Additionally, platinum nanoparticles will achieve both enhanced catalytic surface area and selectivity, which will be beneficial for future glucose monitoring with nano-biosensors.^50^ A 2 µm glucose-specific nanorobot biosensor operates in the bloodstream for 3 months, then self-degrades for immune clearance. Replacing 1,100 annual blood tests, it enables wireless diabetes monitoring with quarterly injections, ensuring durability, selectivity, and compliance with biomedical safety for efficient data collection.^51^

###  AI in insulin delivery

 For patients with type 1 diabetes (PwD), an ideal treatment would mimic natural insulin secretion by using artificial technology (Figure 2). Automated insulin delivery (AID) systems aim to provide this by combining three main components: (1) a continuous glucose monitor (rtCGM) for real-time tracking of glucose levels, (2) a system, like an insulin pump, for precise insulin delivery as needed, and (3) an algorithm that calculates the correct insulin dose based on glucose data from the rtCGM. These algorithms typically run on a smartphone or directly on the insulin pump, which has the necessary processing and display capabilities, as shown in Figure 2.^52^

 Optimal insulin dosing is challenging due to delays in glucose sensing and insulin absorption. Unlike natural insulin, injected insulin acts peripherally first. Bi-hormonal AID systems, or “Bionic Pancreas,” combine insulin and glucagon to improve regulation, but require stable glucagon formulations for effective and practical use. ^53^

 AID algorithms not only consider current glucose levels but also predict future changes to keep glucose in a safe range (70-180 mg/dL). Over recent decades, various algorithms have been developed for AID systems, each with unique strengths and limitations.

 Since the mid-2010s, reliable insulin pumps and rtCGM systems have been available, and algorithm development has progressed as well. In the U.S. and EU, two hybrid AID systems are on the market: the MiniMed 670G by Medtronic (since 2016) and the t:slim X2 CONTROL IQ by Tandem (since 2019).^54^ In the EU, the Diabeloop system, available since 2019, uses a handheld device with an installed algorithm compatible with commercially available insulin pumps (Kaleido) and rtCGMs (Dexcom G6). Diabeloop has also partnered with Roche Diabetes Care to support additional pumps in the future.^32^ The CamAPS FX algorithm, developed at the University of Cambridge and available in the EU since 2020, can be used on Android smartphones with compatible pumps (Soil’s Dana) and rtCGMs (Dexcom G6). Other AID systems, such as the Omnipod 5 from Insulet, are expected to hit the market soon.^55^

###  AI in quality control and quality assurance

 Pharmaceutical quality control is being revolutionized by AI, especially in the area of drug release prediction, as shown in Figure 2. Tablet geometry, drug loading, and compaction pressure are some of the factors that affect drug release, which is evaluated through in vitro and in vivo studies and is crucial in the development of new products.^56^ Drug release research can be complicated and time-consuming using traditional spectrophotometric and analytical techniques. Accurate predictions of dissolution profiles, drug release rates, and disintegration times are now possible thanks to AI techniques like ANN, SVM and regression analysis.^56^

 Quality control is greatly improved when AI and ML are incorporated into Six Sigma documentation. ML algorithms can use predictive analytics to examine past data in order to spot patterns and anticipate possible quality problems, allowing for proactive interventions and risk reduction. By tracking usage trends, predictive maintenance also aids in anticipating equipment failures.^57^ One of the top pharmaceutical companies used ML models and AI-powered data collection to automate the documentation process, track important quality metrics, and identify possible deviations early. Data integrity and compliance were also guaranteed by AI-driven validation checks. This strategy promoted operational excellence and continuous improvement by increasing efficiency, lowering compliance risks, and fortifying overall quality assurance.^57^

###  AI in personalized medicine

 Personalized medicine tailors’ prevention and treatment based on an individual’s molecular, physiological, ecological, and behavioural traits. Unlike standardized care, it uses specific medical data to design precise strategies for disease management and improved overall health outcomes, as shown in Figure 2. The amalgamation of AI with precision medicine has yielded a paradigm shift, as it utilizes sophisticated computational methodologies to analyze medical and family history, as well as genomic information, and electronic health records (EHRs). The capacity of AI to scrutinize these datasets exceeds human proficiency in discerning correlations among disparate datasets, thereby furnishing profound insights into the etiology of diseases and their therapeutic interventions.

 ML constitutes a transformative technological advancement that has fundamentally altered the methodologies employed for the analysis and interpretation of large-scale datasets within the precision medicine, consequently facilitating the personalization of therapeutic strategies. AI assumes a diverse array of functions within precision medicine, tackling numerous critical domains as shown in Table 4.

**Table 4:** Examples of some area where AI plays role in the development of personalised medicine.

**Parameter**	**Description**
Genomic data analysis	Genomic data analysis is a pivotal area where AI demonstrates its potential, as understanding a disease's genetic profile for creating targeted treatment plans. ML and DL technologies enable the analysis of vast datasets to identify mutations, gene expressions, and genetic variations associated with various diseases.^58^
Personalized treatment planning	AI enhances personalized treatment by analyzing genetics, clinical data, and patient responses to recommend tailored therapies. For example, in cancer care, algorithms assess tumour genetics to identify effective treatments, improving outcomes and minimizing side effects based on individual health profiles.^58^
Real-time monitoring and adjustments	AI enables real-time monitoring through wearables and health apps, analysing data to detect complications or poor treatment responses. This allows timely care adjustments—like optimizing insulin doses for diabetics—improving outcomes through personalized, responsive healthcare management.^58^
Integration of multimodal data	AI applied to multisource data—genetic, clinical, imaging, and lifestyle—enhances disease understanding and screening. By linking genomic data with medical imaging, AI reveals correlations between genetic variations and disease traits, improving diagnosis and guiding more effective, personalized treatments.^58^

###  AI in drug repurposing

 Drug repurposing, often called drug repositioning, drug reprofiling or re-tasking, is the process of giving an existing or investigational medication, even those that might be found not approved for the original indication, as shown in Figure 2.The safety profile of many medications is usually known prior to repurposing can potentially reduce drug development timelines and costs to get drugs to patients.^59^ Virtual screening uses AI to repurpose approved drugs by rapidly analysing vast chemical and biological data. Techniques like DL, NLP, and predictive modelling help extract insights from complex datasets. This enhances drug-target interaction prediction, reveals novel drug-disease links, and streamlines the drug repurposing process with greater speed and accuracy.^60^

 The study evaluated applying ML models to repurpose 180 pre-approved drugs with potential efficacy for Pitt–Hopkins syndrome (PTHS). The Prestwick chemical library was screened to identify 55 Kv7.1 and 93 Nav1.8 inhibitors, which we identified as lead therapeutics for potential PTHS intervention.^60^ We then compared our results to a Bayesian ML model’s prediction in Assay Central with the Bayesian ML model identifying 35 Kv7.1 and 64 Nav1.8 inhibitors. The study illustrates how the combined capacity of high-throughput screening (HTS) and ML can be a useful framework for drug repurposing, especially when exploring therapies for rare diseases.^61^

## Ethical and regulatory considerations

 The overview of patient data management, drug development, and clinical decision-making. While these technologies have the potential to improve the accuracy and speed of medical research and treatment, they also raise complex ethical and regulatory challenges.^62^

 The regulation of AI in healthcare has been on the validation of these technologies within strict legal and scientific standards and is monitored strictly. The U.S. Food and Drug Administration (FDA) and the European Medicines Agency (EMA) have published comprehensive guidance on the safe adoption of digital tools, including Software as a Medical Device (SaMD), to ensure their performance and trustworthiness throughout their use in healthcare workflows and drug development.^63^ The Important frameworks such as Good Practice (GxP) and evaluation protocols outlined by these agencies is crucial to secure the accuracy and reliability of AI-driven applications before they are embedded into clinical practice.

 Emphasizing of the World Health Organization (WHO) globally, the need for robust oversight, ethical integrity, and fairness in the application of AI to health that guides and highlights the necessity for transparency, accountability, and inclusive development processes to safeguard public trust and equity during the digital transformation of health systems.^64^ In case of digital healthcare, the priority is to protect the patient privacy. The AI systems routinely process vast repositories of sensitive personal health information. Regulations such as the European General Data Protection Regulation (GDPR) and the U.S. Health Insurance Portability and Accountability Act (HIPAA) establish stringent requirements for managing consent, minimizing data use, and preventing unauthorized access, making it mandatory for organizations to implement strong safeguards throughout the lifecycle of patient data.

 There are high chances of data breaches which is a major concern apart from ethical guidelines which also involves the possible introduction and amplification of biases by AI models. Inaccurate or unrepresentative datasets can skew predictions, leading to care disparities and perpetuating inequities within the healthcare system an issue that has prompted calls from policymakers and global health authorities for routine audits and the use of diverse datasets in AI model development.

 Few of the major tools those are required to improve the model transparency, explain ability tools such as SHapley Additive exPlanations (SHAP) and Local Interpretable Model-agnostic Explanations (LIME) have become essential. These methods allow both practitioners and regulators to understand which features most influence an algorithm’s decision-making process, supporting responsible clinical deployment and regulatory compliance.^65,66^

## Future of AI in pharma world

 AI is rapidly transforming medicine through data integration, aiding healthcare delivery and drug development. Its collaboration with pharmaceutical firms enhances patient care. Notable examples include DeepMind’s partnership with the NHS on kidney injury and the UK’s 100,000 Genomes Project, involving Roche, Merck, Berg, and Biogen, to apply AI in rare disease research. Atomwise is recognized as a leader in healthcare AI, using DL for the first time to discover new small molecule drugs. Benevolent AI is another form of AI now being used in drug development. Numerate concentrates on ligand chemistry, ADMET, and combinatorial ML in conjunction with traditional approaches, placing particular emphasis on the transformation of novel medicinal discovery by addressing significant therapeutic voids through the analysis of extensive datasets related to drug development via algorithmic applications. These improvements are expected to significantly facilitate healthcare services, stratified medicine, clinical trial efficiency, and other domains. At present, the estimated cost of successfully launching a pharmaceutical product onto the market is approximately US$3 billion covering a 15-year time frame.

## Conclusion

 In summary, implementing AI in pharmacy may totally reform the profession and yield significant benefits for patients and pharmacists alike. It may improve medication management, enhance patient outcomes, facilitate drug discovery, and accelerate new drug development through greater accuracy and safety in medication administration. AI-directed advancements may also help decrease healthcare costs. While there are some hurdles facing AI adoption in the pharmacy sector, its potential to transform the pharmacy space is clear.

 AI has impacted numerous areas in the pharmaceutical sector, including drug discovery and development, as well as manufacturing and QA. Where we have seen major changes, mainly in terms of speeding up processes, making them more efficient, and improving outcomes even just as a result of the volume of data that can now be processed, accelerated, and analysed, the ability to recognize and generate patterns and make assumptions from data and enhance decision making with data, and personalization in individual therapies, have certainly been a large part of the overall impact of AI. It has also assisted regulatory compliance, data security, and intellectual property areas in which there are still issues to be addressed, and enhance ongoing efforts in each of these areas.

 Overall, I feel that AI will play a significant role in the advancement of the pharmaceutical industry, innovation and development, and improving outcomes for patients in the future, and it is likely to shape within the next decade, the overall future of healthcare too.

## Competing Interests

 The authors declare no conflict of interest.

## Consent for Publication

 The authors declare no conflict of interest.

## Data Availability Statement

 Data sharing not applicable to this article as no datasets were generated or analysed during the current study.

## Ethical Approval

 Not applicable.
